# Foam Cell Specific LXRα Ligand

**DOI:** 10.1371/journal.pone.0057311

**Published:** 2013-02-25

**Authors:** Radmila Feldmann, Anne Geikowski, Christopher Weidner, Annabell Witzke, Vitam Kodelja, Thomas Schwarz, Mario Gabriel, Thomas Erker, Sascha Sauer

**Affiliations:** 1 Otto Warburg Laboratory, Max Planck Institute for Molecular Genetics, Berlin, Germany; 2 Department of Biology, Chemistry, and Pharmacy, Free University of Berlin, Berlin, Germany; 3 Department of Life Sciences and Technology, Beuth University of Applied Sciences, Berlin, Germany; 4 Department of Medicinal Chemistry, University of Vienna, Vienna, Austria; University of Bari & Consorzio Mario Negri Sud, Italy

## Abstract

**Objective:**

The liver X receptor α (LXRα) is a ligand-dependent nuclear receptor and the major regulator of reverse cholesterol transport in macrophages. This makes it an interesting target for mechanistic study and treatment of atherosclerosis.

**Methods and Results:**

We optimized a promising stilbenoid structure (STX4) in order to reach nanomolar effective concentrations in LXRα reporter-gene assays. STX4 displayed the unique property to activate LXRα effectively but not its subtype LXRβ. The potential of STX4 to increase transcriptional activity as an LXRα ligand was tested with gene expression analyses in THP1-derived human macrophages and oxLDL-loaded human foam cells. Only in foam cells but not in macrophage cells STX4 treatment showed athero-protective effects with similar potency as the synthetic LXR ligand T0901317 (T09). Surprisingly, combinatorial treatment with STX4 and T09 resulted in an additive effect on reporter-gene activation and target gene expression. In physiological tests the cellular content of total and esterified cholesterol was significantly reduced by STX4 without the undesirable increase in triglyceride levels as observed for T09.

**Conclusions:**

STX4 is a new LXRα-ligand to study transcriptional regulation of anti-atherogenic processes in cell or *ex vivo* models, and provides a promising lead structure for pharmaceutical development.

## Introduction

Atherosclerosis and cardiovascular diseases have become enormous health problems during the last decades. Macrophages play a pivotal role in the development and progression of atherosclerosis [1]. A key event of atherosclerosis involves the uncontrolled uptake of oxidized low density lipoproteins (oxLDL) in macrophages, which agglomerate at the subendothelial space of blood vessel walls [2]. When macrophages fail to restore their cellular cholesterol homeostasis via regulate reverse cholesterol transport (RCT) they form diseased foam cells, main components of fatty streaks [3]. The ligand-depend transcription factors LXRα and LXRβ act as cholesterol sensors and respond to elevated oxysterol levels by activating target genes with impact on cholesterol metabolism and atherosclerosis [4]. Many approaches have been pursued to identify a promising LXR ligand with exclusively beneficial properties [5], [6]. Unfortunately, LXRs are also implicated in other counteracting physiological processes, such as triglyceride synthesis [7]. A current challenge is to develop powerful and selective LXR modulators as biochemical tools for mechanistic studies on LXR biology, and to provide pharmaceutically exploitable compounds without mentioned adverse side-effects [8].

While in the past selective agonists for the ubiquitously expressed subtype LXRβ showed in general low efficiency to counteract atherogenic processes, recent evidence suggests that rather the LXRα subtype plays a crucial role for regulating anti-atherogenic gene expression profiles [9]. In human macrophages LXRα is regulated via a feed-forward autocatalytic loop leading to significantly increased levels by LXRα ligand-treatment [10]. In mouse macrophages this effect was not observed, indicating that activation in human foam cells differs strongly from mouse foam cells.

Here we introduce an LXRα ligand that specifically activates target genes in diseased human foam cells but not in macrophages. This activation resulted in significant reduction of total and esterified cholesterol with similar potency as for the synthetic LXRα/β ligand T0901317 (T09) - without the observed counteracting increases of triglyceride levels known from previously published LXRα/β ligands.

## Materials and Methods

### LXRα Ligand Screening

We used the LXR alpha coactivator kit (Invitrogen) and screened a biologically diverse library of >7,000 compounds for nanomolar binders according to manufacturer’s instructions. T0901317 (T09) was obtained from Sigma-Aldrich.

### STX4 Ligand Synthesis

All chemicals were obtained from commercial suppliers and used without further purification. ^1^H and ^13^C NMR spectra were recorded on a Bruker Advance DP×200. Chemical shifts values are reported in ppm relative to Me_4_Si. TLC was performed on MERCK silica gel TLC aluminium sheets with fluorescent indicator 254 nm. Elemental analysis was performed on a Perkin Elmer 2400 CHN Elemental Analyzer at the microanalytical laboratory of the University of Vienna. Mass spectra were performed on a Shimadzu (GC-17A; MS-QP5050A) spectrometer.

For synthesis of 4-[(E)-styryl]phenol (compound 1) we used a mixture of 4-hydroxybenzaldehyde (0.244 g; 2.00 mmol), phenyl acetic acid (0.272 g; 2.00 mmol) and piperidine (catalytic amount) and heated all at 240°C for 15 minutes. After cooling to room temperature 40 ml of ethyl acetate was added. The organic phase was washed with water and dried with sodium sulfate. The solvent was evaporated under reduced pressure and the product purified by chromatography (petroleum ether/ethyl acetate 8∶2) to obtain title compound as white solid.

Mp: 184–186°C; Yield: 31.6%; ^1^H NMR (*d_6_*-DMSO, 200 MHz): δ 6.71–6.88 (m, 2H, ArH), 6.94–7.63 (m, 9H, ArH), 9.30–9.88 (bs, 1H, OH). ^13^C NMR (*d_6_*-DMSO, 50 MHz): δ 115.6 (2C, CH), 125.1 (1C, CH), 126.0 (2C, CH), 127.0 (1C, CH), 127.9 (2C, CH), 128.1 (1C, C_q_), 128.4 (1C, CH), 128.6 (2C, CH), 137.5 (1C, C_q_), 157.3 (1C, C_q_). Anal. Calcd. for C_14_H_12_O: C, 85.68; H, 6.16. Found: C, 85.59; H, 5.97. MS *m/z* 196 (100%, M^+^), 195 (39%), 177 (31%), 165 (32%).

For further synthesis of 2-[[4-[(E)-styryl]phenoxy]methyl]oxirane (STX4) we used a suspension of compound 1 (0.196 g; 1.00 mmol) and NaOH (0.048 g; 1.20 mmol) in epichlorohydrin was heated at 130°C for 6 hours. The mixture was then evaporated under reduced pressure and redissolved in diethyl ether. The precipitate was filtered off and the filtrate was washed with water. The organic layer was dried over sodium sulfate and evaporated under reduced pressure. The dried product was recrystallized from ethanol.

Mp: 133–135°C; Yield: 50.4%; ^1^H NMR (CDCl_3_, 200 MHz): δ 2.72–3.01 (m, 2H, CH_2_), 3.28–3.50 (m, 1H, CH), 3.90–4.35 (m, 2H, CH_2_), 6.84–7.15 (m, 4H, Ar H), 7.20–7.64 (m, 7H, Ar H). ^13^C NMR (CDCl_3_, 50 MHz): δ 44.9 (1C, CH2), 50.2 (1C, CH), 68.9 (1C, CH2), 115.0 (2C, CH), 126.4 (2C, CH), 127.1 (1C, CH), 127.4 (1C, CH), 127.9 (2C, CH), 128.2 (1C, CH), 128.8 (2C, CH), 130.8 (1C, C_q_), 137.7 (1C, C_q_), 158.3 (1C, C_q_). Anal. Calcd. for C_17_H_16_O_2_: C, 80.93; H, 6.39. Found: C, 80.90; H, 6.17. MS *m/z* 252 (100%, M^+^), 196 (49%), 195 (44%), 165 (43%), 152 (40%).

### Cell Culture

We used the human THP1 cell line, which is widely applied as a macrophage and foam cell model with similar properties as primary cells [11]. THP-1, a human acute monocytic leukemia cell line, was obtained from Deutsche Sammlung von Mikroorganismen und Zellkulturen (DSMZ) and cultivated at 37°C (5% CO_2_, 95% humidity) in RPMI 1640 (Invitrogen) supplemented with 10% FBS Superior (Biochrom). For generating macrophages differentiation was induced by 10^−8^ M Phorbol 12-myristate 13-acetate (PMA) (Sigma-Aldrich) for 48 h. Ligand treatment of macrophages was performed for further 24 h. To induce foam cell formation macrophages were treated for additional 48 h with 100 µg/mL modified human oxidized low density lipoprotein (oxLDL) obtained from Source BioScience (RP-048). Ligand treatment was performed simultaneously with foam cell formation. Cholesterol loading and treatment was controlled with Oil Red O staining (Alfa Aeser). To investigate ligand induced effects on cell proliferation of THP-1 macrophages CellTiter-Glo Luminescent Cell Viability Assay (Promega) was utilized.

For dual luciferase reporter gene assay we used human embryonic kidney (HEK-293) cells that were cultivated in Dulbeccós Modified Eagle Medium (DMEM) with 4,5 g/L glucose (Invitrogen) and 10% FBS Superior at 37°C (5% CO_2_, 95% humidity).

### Plasmid Cloning and Dual Luciferase Reporter Gene Assay

The ligand binding domain (LBD) of LXRα (amino acids 164 to 447) or LXRβ (amino acids 154 to 461) was cloned into pBIND [Renilla/Amp] vector (Promega). Selection and amplification were performed in *E. coli* cells. Purification of the plasmids was performed with a plasmid purification kit (Qiagen). For transfection HEK-293 cells were plated in 96-well plates (TPP) at a density of 3.5×10^4^ cells/well for 24 h. To investigate the ligand binding on LXRα ligand binding domain, 0.04 ng of renilla luciferase coding plasmide pGL4.75 (Promega), 0.04 ng of LXRα-LBD/GAL4-DBD plasmide and 80 ng of firefly luciferase coding plasmide pGL4.31 (Promega) were used in a total volume of 200 µL for each well. For LXRβ binding assay 40 ng of LXRβ-LBD/GAL4-DBD plasmide were applied in each transfection mix. Transfection was carried out for 4 h with Lipofectamine 2000 Transfection Reagent (Invitrogen) in Opti-MEM medium (Invitrogen). Ligand treatment was performed at different concentrations for 24 h. For lysis and detection dual-luciferase reporter gene assay system (Promega) and POLARstar Omega (BMG LABTECH) were used.

### LXR Knockdown

Target specificity of ligand-dependent gene expression effects was investigated in siRNA-mediated LXRα and β-knockdown foam cells with subsequent qPCR or microarray analysis. Therefore, 2×10^5 ^THP-1 cells/well were differentiated in 24-well plates and induced to foam cells prior to knockdown as described above. Foam cells were transfected using TransIT-TKO transfection reagent (Mirus Bio), for combined knockdown with 15 nM LXRα Silencer Validated siRNA (Ambion, ID 5458) and 15 nM LXRβ Silencer Select Validated siRNA (Ambion, ID s14684) and with 30 nM LXRα or LXRβ for individual knockdowns, respectively. As control we used 30 nM Silencer Select Negative Control #1 (Ambion). Transfection was carried out for 48 h and followed by treatment with 10 µM ligand or vehicle control for 24 h.

### Real-time Polymerase Chain Reaction

Total RNA isolation and purification was performed by using the RNeasy Mini Kit (QIAGEN) and RNase-Free DNase Set (QIAGEN). cDNA was generated by reverse transcription using the High Capacity cDNA Reverse Transcription Kit (Life Technologies). Gene expression was quantified using the SYBR Green PCR Master Mix (Life Technologies) and ABI 7900HT Fast Real Time PCR System (Life Technologies). In each reaction 0.8 ng/µL cDNA and 0.2 µM primers were used. Gene expression was calculated with the ΔΔCt-method. ß-Actin was used for normalisation.

### Microarray Analysis

Microarray analyses were performed according to instructions of Illumina‘s TotalPrep RNA Amplification Kit followed by hybridisation on HumanHT-12 v3 or HumanHT-12 v4 Expression BeadChips (Illumina). Data analysis was performed with GenomeStudio V2011.1 (Illumina). Differential expression analysis was performed on background-subtracted data with cubic spline normalization and Benjamini-Hochberg FDR correction. Significant data was considered to have a detection p-value of ≤0.01 and differential expression p-value of ≤0.05 according to Illumina’s t-test error model. Gene expression data were submitted in MIAME-compliant form to the NCBI Gene Expression Omnibus database (GSE39079) [12]. Bead array data were validated by quantitative real-time PCR. The Gene Distance Matrix was conducted in Euclidean space using the MeV 4.3 software tool [13]. Gene Set Enrichment Analysis (GSEA) was performed as described elsewhere [14] with the following parameters: 1000 gene set permutations, weighted enrichment statistics, minimal gene set size of 5, and signal-to-noise metric. Enrichment of pathways was tested using the Reactome (v3.0) and the KEGG (v3.0) database from the Molecular Signature Database (MSigDB). Heatmaps were carried out with Mayday 2.8 [15]. For presentation of treatment effects in the heatmap, the pathway normalized enrichment score (NES) was adjusted with the appropriate FDR as follows: adjustedNES = (1−FDR)×NES.

### Western Blotting

Whole cell extracts were harvested from three biological replicates and denaturized for SDS-PAGE (NuPAGE Novex 4–12% Bis-Tris Gels). Proteins were blotted on nitrocellulose Hybond ECL membrane and incubated with strictly validated published antibodies against LXRα (Abcam, ab 41902), APOE (Abcam ab1906), and as housekeeping protein control β-Actin (Santa Cruz Biotechnology, sc-47778, C4). Secondary antibodies were HRP labeled anti-mouse and anti-rabbit (Santa Cruz Biotechnology). After detection with Western Lightning Plus-ECL solution membranes were stripped with Restore Plus Western Blot Stripping Buffer. Densitometry was performed in ImageQuant TL (GE Healthcare). This tool measures quantitatively the optical density and provides more accurate data than simple eye-observations. The calculation of log (protein of interest/β-Actin) corrects data for loading imbalances.

### Cholesterol and Triglyceride Analyses

THP-1 monocytes were plated out into 24-well plates (Nunc) at a density of 2×10^5^ cells/well. After differentiation and treatment cells were lysed with 100 µL lysis buffer (PBS, 0.25 M NaCl, 1% Triton X-100). Total and free cholesterol were determined using the colorimetric enzymatic Amplex Red Cholesterol Assay Kit (Invitrogen). Triglycerides were quantified by Triglyceride Assay Kit (BioVision). For normalisation protein content was determined with Pierce 660 nm Protein Assay Reagent (Thermo Scientific).

### Statistical Analyses

Statistical significance was determined by unpaired two-tailed Student’s t-test for single comparisons and one-way ANOVA with Dunnett’s post-hoc test for multiple comparisons. Statistical analyses were carried out using GraphPad Prism 5.0. A p-value ≤0.05 was defined as statistically significant.

## Results

**Figure 1:**
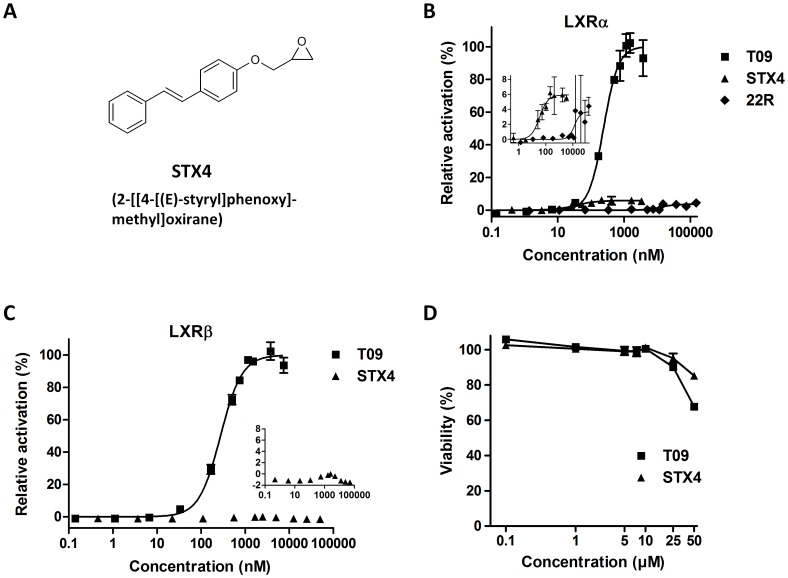
STX4 is a novel selective LXRα agonist. **A**, Chemical structure of STX4. **B**, Transcriptional activation of LXRα by T0901317 (T09), 22-R-hydroxycholesterol or STX4 in a reporter gene assay (see also Table 1). Data are expressed as mean±SD (n = 3). **C**, Validation of LXRα specificity. Transcriptional activation of LXRβ by T09 or STX4 in a reporter gene assay. STX4 does not activate the LXRβ subtype (mean±SD, n = 3). **D**, Cytotoxicity of STX4 in macrophages (mean±SD, n = 3). STX4 does not reduce cellular viability up to 25 µM.

During the screening of a natural products library [16], we identified the stilbenoid backbone as a suitable candidate structure for activating LXR. Thereby, we observed an increased activation potential of stilbenoids with an epoxide (STX4, Figure 1A). To analyse binding and activation ability of the new ligand STX4 we applied dual luciferase reporter-gene assays with LXRα and LXRβ ligand binding domains (LBD) in HEK-293 cells (Figure 1B and Figure 1C). Whereas the control ligand T09 bound to LXRα and LXRβ with similar affinity, STX4 showed characteristic specificity for LXRα (EC_50_ = 35 nM) but less efficient transcriptional activation (6% vs. T09). We additionally measured the LXRα activation potential of the natural ligand 22-R-Hydroxycholesterol (EC_50_ = 13.3 µM, Figure 1B) and observed even lower transcriptional activation (4% vs. T09).

As for T09, viability assays revealed no cytotoxic effects of STX4 up to 50 µM (Figure 1D), making this compound suitable for cell culture studies.

**Figure 2:**
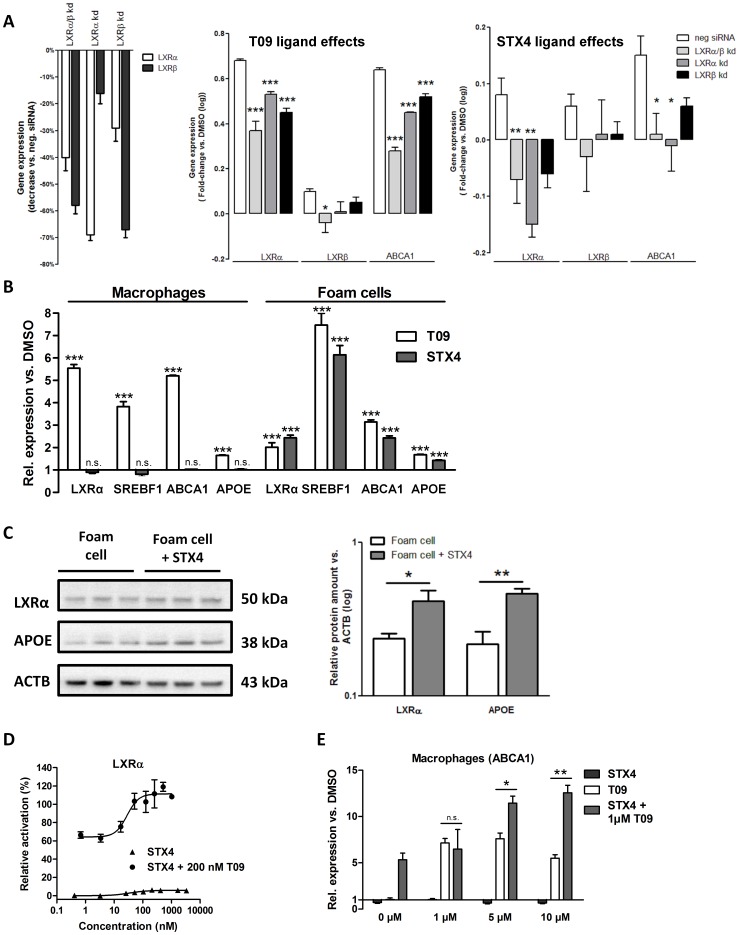
STX4 specifically targets diseased, oxysterol-loaden foam cells. **A**, Gene expression in foam cells after siRNA-mediated, single and combined LXRα/β knockdown (mean±SEM, n = 4, fold-change vs. DMSO, logarithmised). Left, efficiency of LXRα, LXRβ and LXRα/β knockdown (kd). Middle, LXRα, LXRβ and LXRα/β knockdown (kd). Middle, single and combined knockdown influence on gene expression of LXRα, LXRβ and ABCA1 upon T09 treatment. Right, single and combined knockdown influence on gene expression of LXRα, LXRβ and ABCA1 upon STX4 treatment. *P<0.05, **P<0.01, ***P<0.001 vs. negative siRNA. **B**, Gene expression in THP-1 macrophages and foam cells after treatment with T09 (10 µM) or STX4 (10 µM). Data are expressed as mean±SEM (n = 4). **C**, Western blot analysis of LXRα and APOE content in foam cells and STX4 treated foam cells. Bar plot displays the results of densitometry analysis (mean±SEM, n = 3). *P<0.05, **P<0.01 vs. foam cell.**D**, Transcriptional activation of LXRα by STX4 in the presence or absence of 200 nM T09 (see also table **1**). Data are expressed as mean±SD (n = 3). **E**, Gene expression in THP-1 macrophages after treatment with different concentrations of T09 or STX4 in the presence or absence of 1 µM T09. Data are expressed as mean±SEM (n = 2–3). *P<0.05, **P<0.01, ***P<0.001 vs. DMSO; n.s., not significant.

To test for ligand specificity we performed LXRα and LXRβ knockdown experiments (Figure 2A). For the T09 ligand we detected in absence of both subtypes a significant reduction of LXRα and ABCA1 expression, whereas STX4 treatment showed a higher dependency on the LXRα subtype. Notably, the STX4 knockdown study was less robust than with T09 due to apparent cross-reactivity of STX4 with the transfection reagent. However, consistent with the above described reporter gene assays we observed in foam cells higher LXRα than LXRβ specificity of STX4.

For testing the activation of LXRα in human macrophages and in diseased human foam cells we performed gene expression analyses of central LXRα target genes (namely *LXRα* itself, *SREBF1, ABCA1,* and *APOE* in Figure 2B). In macrophages we observed increased expression of LXRα target genes upon T09 treatment, but not with STX4. Strikingly, in foam cells STX4 treatment resulted in potent target gene expression similar to T09, which was consistent with increased protein expression levels of LXRα target genes (Figure 2C).

In a competitive reporter-gene assay with a fixed concentration of T09 and increasing levels of STX4, we detected additive effects on reporter gene activation (efficacy range 64%–111%, Figure 2D, Table 1) and *ABCA1* expression in macrophages (Figure 2E), indicating conditional activation potency of STX4 in foam cells. These data suggest that activation of LXRα upon STX4 treatment occurred selectively under the diseased condition of the foam cell.

**Table 1:** Results from competitive reporter-gene assays.

Compound	+DMSO		+200 nM T0901317 (additive)	
	EC_50_	Efficacy	EC_50_	Efficacy(range)
**T0901317**	239 nM	100%		
**22-R-Hydroxycholesterol**	13.3 µM	4%		
**STX4**	35 nM	6%	28 nM	47%(64 to 111%)

**Figure 3:**
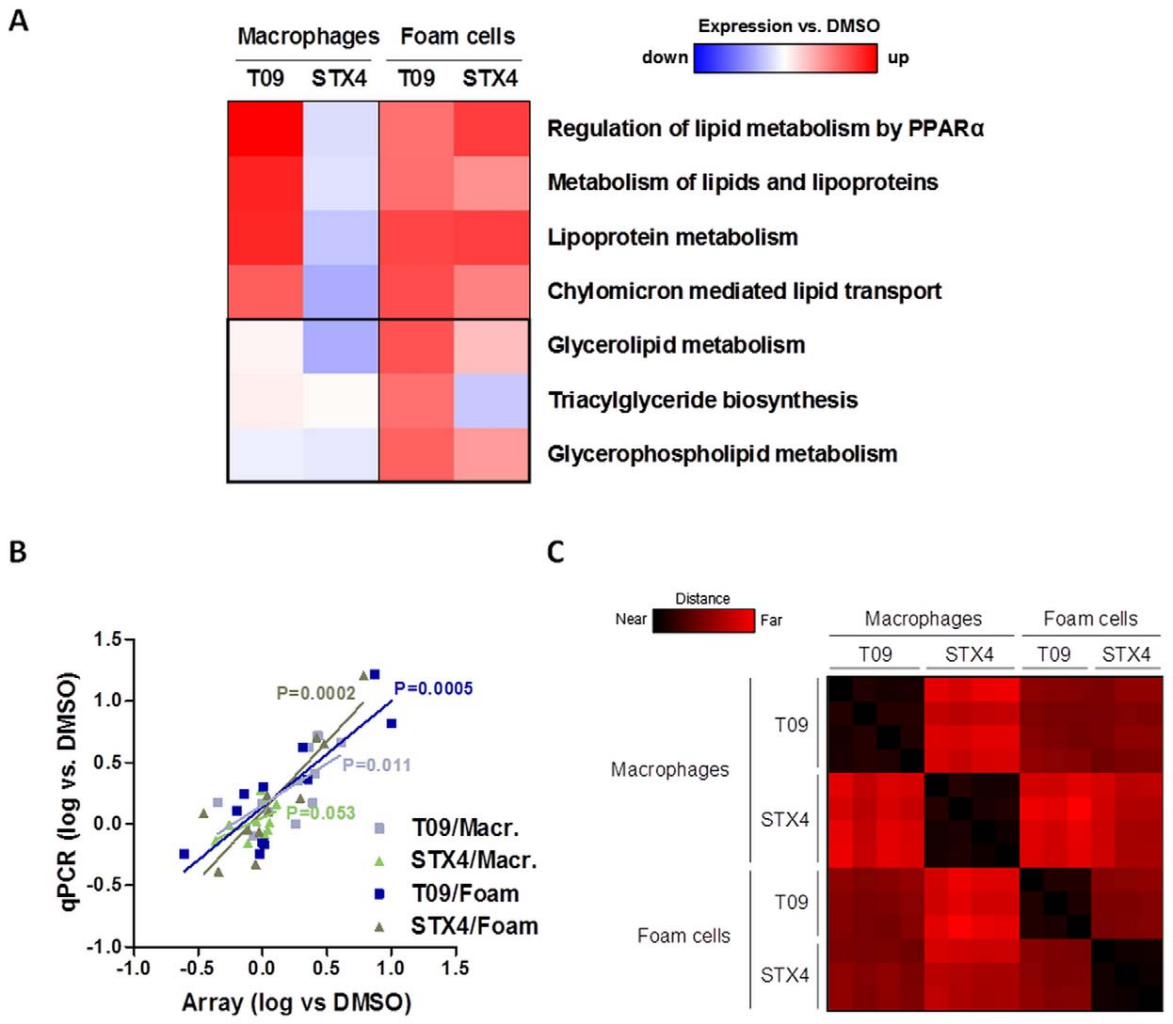
STX4 specifically regulates gene expression in foam cells. **A**, Genome-wide gene expression and subsequent gene set enrichment analysis (GSEA) of macrophages and foam cells after treatment with T09 (10 µM) or STX4 (10 µM). Regulation of lipid-derived Reactome and KEGG pathways is shown. **B**, Validation of gene expression microarray analysis by quantitative PCR. For all tested samples, array observations significantly correlated with qPCR results. **C**, Gene distance matrix of genome-wide gene expression analysis of macrophages and foam cells after treatment with DMSO (0.1%), T09 (10 µM) or STX4 (10 µM). Pairwise distances were calculated for comparison of two treatments and cell types. Colored squares show the distance in Euclidean space, ranging from exactly the same profile (black) to completely different (red). In macrophages STX4 expression is very different to that of T09 (P<0.0001), whereas in foam cells STX4-induced gene expression is similar to that of T09 (P<0.0001).

**Figure 4:**
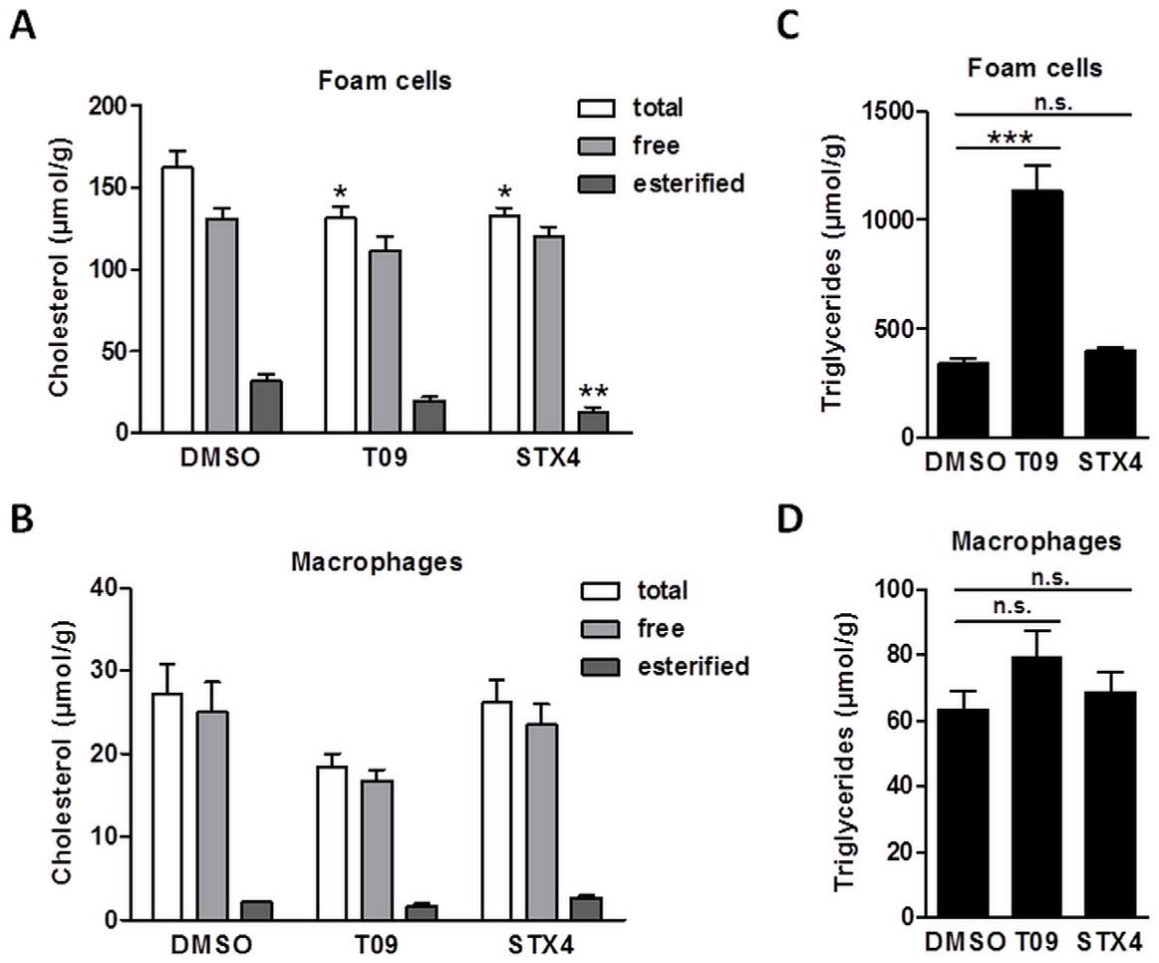
Physiological analyses of STX4 treatment confirm unique STX4 properties in foam cells. **A**, Cholesterol content in foam cells after treatment for 48 h with DMSO (0.1%), T09 (10 µM) or STX4 (10 µM). Data are expressed as mean±SEM (n = 5–6). **B**, Cholesterol content in macrophages after treatment for 48 h with DMSO (0.1%), T09 (10 µM) or STX4 (10 µM). Data are expressed as mean±SEM (n = 5–6). **C**, Triglyceride content in foam cells after treatment for 48 h with DMSO (0.1%), T09 (10 µM) or STX4 (10 µM). **D**, Triglyceride content in macrophages after treatment for 48 h with DMSO (0.1%), T09 (10 µM) or STX4 (10 µM). Data are expressed as mean±SEM (n = 5–6). ***P<0.001 vs. DMSO; n.s., not significant.

To study the genome-wide effects of STX4 treatment we performed microarray-based expression analyses on STX4-, T09- or DMSO-treated macrophages and foam cells (Figure 3A). Consistent with qPCR data (Figure 3B), expression of genes involved in lipid metabolism processes was activated in T09-treated but not in STX4-treated macrophages. However, in foam cells we found a high similarity between STX4 and T09 treatment (Figure 3C). Lipid metabolism processes were comparably up-regulated by T09 and STX4 in foam cells (Figure 3A), which consequently resulted in a significant reduction of total and esterified cholesterol content in contrast to macrophage treatments (Figure 4A and Figure 4B).

Interestingly, we noted a difference between T09 and STX4 treatments in terms of triacylglyceride biosynthesis. While this process was transcriptionally induced by T09 treatment, it was not regulated by STX4 treatment in foam cells (Figure 3A). Consistently, treatment with T09 but not STX4 led to increased triglyceride levels in foam cells (Figure 4C). In macrophages the triglyceride level stayed rather unchanged for both treatments (Figure 4D).

Notably, despite the observed up-regulation of *SREBF1* mRNA in STX4-treated cells (Figure 2B), which is a common marker gene for lipogenesis, triglyceride levels were not increased. This example illustrated the strength of the applied gene set enrichment analysis method to decipher functional pathways and explain complex effects of nuclear receptor ligands compared to limited information from conventional single gene centered assessments. Further studies are needed to explore the observed effects. Anyway, the LXRα agonist STX4 featured a high potential to reduce excess cholesterol without undesirable increase in triglycerides in foam cells.

In summary, STX4 is a foam cell specific gene regulating molecule, which showed no significant activity in normal macrophages. This new LXRα ligand can be applied for investigating mechanistic aspects of macrophage homeostasis during (anti-) atherogenic processes, and can be used as a lead structure for pharmaceutical development.

## Discussion

Specific ligands for LXRs are important tools to combat atherosclerosis and cardiovascular diseases [17]. In this study we introduced the new LXRα-specific ligand STX4, which has a stilbenoide structure that is derived from the natural LXR ligand oxysterol or sterols in general.

Our data suggest that the unique conditional activation potential of STX4 in diseased foam cells depends on a partner ligand. As we could show with reporter-gene assays and gene expression analyses, STX4 cooperates with the synthetic ligand T09 as well as with natural ligands derived from oxLDL in an additive manner. An explanation for this behaviour could be allosteric binding of STX4 to the ligand binding domain. In addition we assume that a changed set of transcriptional LXR co-factors in foam cells could be recruited by STX4 and thereby drive its specific actions. Due to its autoregulatory activation potential, LXRα has highly increased protein content in foam cells compared to macrophages [10]. Strikingly, STX4 was not efficient in activating LXR-target genes in macrophages but only in foam cells. Lipid loaded foam cells may provide a chemically favourable hydrophobic, fatty acids rich environment for the STX4 compound. Moreover, the level of LXRα in foam cells in our experiments was at least 10-fold higher than for LXRβ (data not shown), suggesting that the observed effects of STX4 were mainly driven by the alpha-subtype. This hypothesis could be confirmed in reporter gene assays and by trend in LXR knockdown experiments in cell culture (Figures 1B–C and 2A).

The discussed molecular interaction of STX4 with LXRα could eventually contribute to the observed conditional activation of LXRα pathways in diseased foam cells. The STX4 molecule can be readily applied in cell culture and *ex vivo* models to study gene regulation processes and resulting metabolic effects.

Most LXRα/β ligands failed as therapeutic targets due to a lack of target gene activation potency or adverse side-effects such as lipogenesis. Due to its more specific anti-atherogenic spectrum the foam cell specific ligand STX4 holds promise for further pharmaceutical development. This work will comprise chemical optimization and modification of the presented STX4 lead structure, testing of chemical stability and potential side effects including for example binding tests with nuclear receptors and other regulating proteins, and applying test panels including absorption, distribution, metabolism and excretion (ADME), prior *in vivo* analyses in model animals. However, the autoregulation of ligand-activated LXRα in human foam cells varies significantly from mouse foam cells [10], showing one of the severe limitations of using mouse models for studying the physiological effects of drug candidates for treating atherosclerosis [2].

As exemplified in this study, the development of potent compounds for specifically activating key metabolic nuclear receptors in diseased but not in normal target cells provides new avenues for mechanistic studies and for innovative treatment strategies.
